# Multifaceted mechanisms mediating cystine starvation-induced ferroptosis

**DOI:** 10.1038/s41467-021-25159-5

**Published:** 2021-08-09

**Authors:** Zhennan Shi, Nathchar Naowarojna, Zijian Pan, Yilong Zou

**Affiliations:** 1 Westlake Four-Dimensional Dynamic Metabolomics (Meta4D) Lab, Westlake Laboratory of Life Sciences and Biomedicine, Hangzhou, Zhejiang China; 2 School of Life Sciences, Westlake University, Hangzhou, Zhejiang China; 3 Institute of Biology, Westlake Institute of Advanced Studies, Hangzhou, Zhejiang China

**Keywords:** Lipid peroxides, Cancer metabolism, Necroptosis, Target identification

## Abstract

The cyst(e)ine/glutathione (GSH)/glutathione peroxidase 4 (GPX4) axis is the most frequently targeted pathway to trigger the ferroptosis cascade and suppress tumor growth. Two recent studies present additional mechanisms underlying cystine starvation-induced ferroptosis apart from impaired GSH synthesis.

Ferroptosis is an iron-dependent cell-death modality driven by aberrant accumulation of peroxidized polyunsaturated phospholipids^1^. Ferroptosis has been widely observed in small-molecule treated cells in vitro, and was implicated in various pathological conditions including neurodegeneration, ischemia/reperfusion-induced kidney, heart and liver injury, and stroke^2^. This cell death modality was also proposed as a potential therapeutic target for treating multiple cancers including kidney, ovarian, liver, and pancreatic cancers^3^. Hence, both bioavailable ferroptosis inhibitors and inducers are highly desirable for treating human diseases.

While the proteins and metabolites that directly participate in ferroptosis execution remain under search, recent studies highlighted multiple pathways that together keep spontaneous lipid peroxidation below the detrimental threshold and suppress ferroptosis. These pathways include the cyst(e)ine/glutathione (GSH)/glutathione peroxidase 4 (GPX4) axis^1,4^, the ferroptosis suppressor protein 1 (FSP1)/coenzyme Q_10_ (CoQ_10_) axis on the plasma membrane^5,6^, the guanosine triphosphate cyclohydrolase 1 (GCH1)/tetrahydrobiopterin (BH4)/dihydrofolate reductase (DHFR) axis^7,8^, and the mitochondrial dihydroorotate dehydrogenase (DHODH)/CoQ system^9^. Currently, blocking these endogenous ferroptosis-suppressive pathways is the most frequently adopted strategy for ferroptosis induction. GPX4 is the only cellular enzyme that specifically reduces phospholipid hydroperoxides to lipid alcohols using GSH as a co-factor^4^, and inhibiting the cyst(e)ine/GSH/GPX4 axis induces the strongest cell death in most cellular contexts.

Within the cyst(e)ine/GSH/GPX4 axis, cysteine is the rate-limiting metabolite for GSH biosynthesis, hence the ferroptosis-inducing activity of cyst(e)ine depletion has largely been attributed to the lowering of intracellular GSH levels and subsequent decrease of GPX4 activity. However, cysteine depletion generally induces stronger ferroptotic responses compared to GSH depletion^10^, which is suggestive of additional mechanisms underlying cysteine starvation-induced ferroptosis. Recently, Zhang et al. reported that cystine starvation impairs GPX4 protein expression by inhibiting mTORC1/4E-BP1-mediated protein translation^11^. In an earlier study, Kang et al. showed that intracellular glutamate accumulation following cyst(e)ine depletion or system x_C_- inhibition is a key contributor to the induction of ferroptosis, and glutamate toxicity can be alleviated by converting excess glutamate to γ-glutamyl-di- or tripeptides via promiscuous enzymatic activities of glutamate-cysteine ligase catalytic subunit (GCLC)^12^. These two studies pinpointed that the effects of cyst(e)ine depletion are not linear, but rather form an intertwined metabolic network to drive ferroptotic cell death.

Zhang et al. systematically characterized differentially expressed proteins induced by cystine starvation and found that GPX4 was among the most downregulated proteins^11^. This result suggested that cystine starvation may induce ferroptosis by both blocking GSH biosynthesis and reducing GPX4 protein level. Cystine starvation-induced GPX4 protein downregulation was mediated by protein translation suppression rather than reduction in mRNA transcription or protein stability. The authors reasoned that the regulation of GPX4 protein translation might involve amino acid-sensing mechanisms such as mTORC1 signaling. Indeed, under cystine starvation, mTORC1 other than mTORC2 signaling was suppressed; and chemical and genetic inhibition of mTORC1 signaling sensitized cancer cells to ferroptosis. Further characterizations suggested that cyst(e)ine regulates GPX4 expression through the RagA/B-mTORC1-4EBP axis. Though precisely how RagA/B-mTORC1 senses cyst(e)ine levels and how mTORC1 specifically regulates GPX4 protein synthesis remain to be fully characterized. Importantly, combining mTORC1 inhibitors with imidazole ketone erastin (IKE), a bioavailable ferroptosis inducer, exhibited synergistic tumor-suppressing effects in patient-derived xenografts of lung cancer. Together, these findings underscore the bifunctionality of cysteine in regulating protein translation besides serving as the substrate for GSH biosynthesis (Fig. 1).

**Fig. 1:**
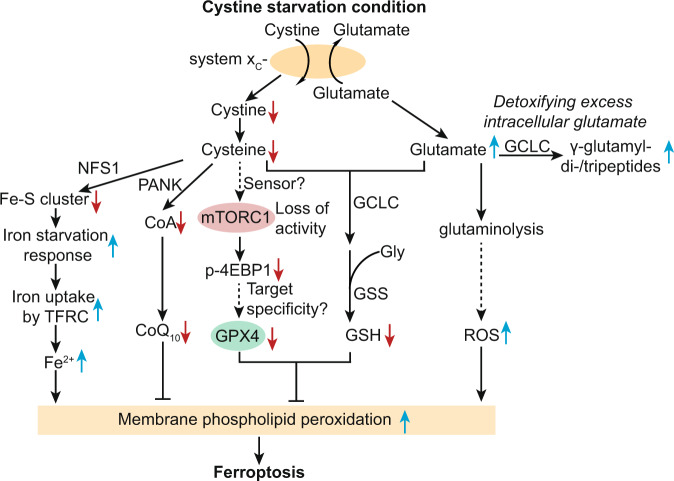
Metabolic pathways driving the ferroptosis-susceptible state in response to cystine starvation in cancer cells. GSH glutathione (reduced), GPX4 glutathione peroxidase 4, GCLC glutamate-cysteine ligase catalytic subunit, GSS glutathione synthetase, TFRC transferrin receptor, NFS1 cysteine desulfurase, Fe–S cluster iron–sulfur cluster, ROS reactive oxygen species, CoA co-enzyme A, PANK pantothenate kinases, 4EBP1 eukaryotic translation initiation factor 4E binding protein 1.

Cellular cystine uptake is mainly mediated by the system x_C_- cystine/glutamate antiporter. Among the metabolic alterations induced by cystine deprivation, one often neglected consequence is the aberrant accumulation of intracellular glutamate due to the lack of extracellular cystine as exchange molecules for sustained system x_C_- activity. How does the imbalanced glutamate pool affect cellular metabolism? Kang et al. reported that a panel of non-small cell lung cancer (NSCLC) cell lines exhibited heterogeneous responses to cystine starvation despite the consistent intracellular cysteine deletion and impaired GSH biosynthesis^12^. Comparative metabolomic profiling of cystine-repleted or -starved NSCLC cells revealed that cystine-starved cells accumulate γ-glutamyl-di- or tripeptides, which were produced by non-canonical activity of GCLC. The γ-glutamyl-peptide levels were inversely correlated with cystine starvation-induced ferroptosis sensitivity across the NSCLC cell line panel. Although γ-glutamyl-peptides per se did not directly protect cells against ferroptosis under cystine starvation, their biosynthesis consumes glutamate, an intracellular amino acid that was previously implied in ferroptosis induction via glutaminolysis^13^. Does the consumption of glutamate by GCLC protect cells from ferroptosis induced by cystine starvation? The authors leveraged the epistatic relationship between GCLC and glutathione synthetase (GSS) in GSH synthesis. GCLC knockout depletes both GSH and γ-glutamyl-peptides, blocks glutamate consumption, and enhances sensitivity to ferroptosis; in contrast, GSS knockout depletes GSH but still permits glutamate consumption for γ-glutamyl-peptide synthesis, and does not alter ferroptosis sensitivity under cystine starvation. These results, together with a series of complementary experiments, confirmed that the non-canonical activity of GCLC protects cystine-starved cells from ferroptosis in a GSH-independent manner^12^. Collectively, Kang and colleagues reinforced the observation that cystine starvation could sensitize cancer cells to ferroptosis by inducing intracellular glutamate accumulation, and uncovered that GCLC potentially alleviates cellular ferroptotic stress by both mediating GSH synthesis and scavenging glutamate.

In addition to the consensus on the prominent role of cystine starvation in blocking GSH synthesis, the present two studies demonstrated two previously under-appreciated metabolic effects that contribute to the ferroptosis-susceptible state in cancer cells under cystine deprivation conditions: loss of GPX4 protein translation and accumulation of oxidative stress-inducing intracellular glutamate (Fig. 1). These studies highlight the synergistic effects between ferroptosis induction and mTORC1 inhibition or GCLC inhibition as potential cancer treatment. Evidently, intracellular cyst(e)ine contribute to ferroptosis suppression via at least two additional pathways in different contexts: in a mouse lung adenocarcinoma model, NFS1 cysteine desulfurase utilizes cysteine to synthesize iron–sulfur clusters, which are necessary for preventing activation of the iron-starvation response, subsequent iron influx and ferroptosis^14^ (Fig. 1). Moreover, a small fraction of intracellular cysteine is used for the biosynthesis of coenzyme A and subsequently CoQ_10_, a key metabolite for preventing membrane lipid peroxidation and ferroptotic cell death^5,6,15^ (Fig. 1). Taken together, there are at least five metabolic axes essential for cellular redox homeostasis that were interrupted by cystine deprivation. These metabolic axes take part in shaping the ferroptosis-susceptible cell-state collectively, and the relative contribution of each axis is likely dictated by the cellular contexts and by the specific ferroptosis-inducing approaches (Fig. 1).

Findings in these interesting studies also raised several unanswered questions. First, while the Zhang et al. study pinpointed the important role of mTORC1 signaling in regulating ferroptosis sensitivity in cancer cells, the contribution of mTORC1 to ferroptosis and the underlying mechanisms appear highly context-specific. Recent studies in cardiomyocytes^16^ and PI3K-hyperactive human breast and prostate cancer cells^17^ both confirmed that mTORC1 activation protects cells from ferroptosis. Specifically in the PI3K-activated cancer cells, Yi et al. revealed that mTORC1 enhanced ferroptosis resistance by upregulating sterol regulatory element-binding protein 1 (SREBP1)^17^. SREBP1 activation induces stearoyl-CoA desaturase (*SCD1*) expression, which presumably promotes the accumulation of ferroptosis-inhibitory monounsaturated fatty acids^18^ though this connection awaits to be validated by lipidomics. Notably, in a recent chemical screen in the ferroptosis-sensitive HT-1080^N^ fibrosarcoma cells, seven out of eight PI3K/mTOR inhibitors suppressed the ferroptosis-inducing activity of erastin and sorafenib, but not the covalent GPX4 inhibitor, ML162^19^. The contrasting responses to combined mTORC1 inhibition and ferroptosis induction in different cellular contexts are likely dictated by complex factors including genetic mutation landscape, the ratio of polyunsaturated to monounsaturated phospholipids, iron availability, the basal activities of the PI3K/mTORC1 pathway, and the overall cellular redox state. A high-throughput approach that examines large collections of cancer models is likely required to systematically resolve the role of PI3K/mTORC1 in ferroptosis sensitivity in various cancer types.

Second, the precise mechanisms by which elevated intracellular glutamate induces ferroptosis in cancer cells remain poorly understood. In previous studies, excess extracellular glutamate suppresses the antiporter activity of system x_C_- therefore inhibits the uptake of cyst(e)ine and sensitizes cells to ferroptosis^1^. Elevated intracellular glutamine was also implicated in ferroptosis induction via glutaminolysis yet the chemical processes that lead to increased cellular oxidative stress await to be dissected^13^. Metabolic flux analysis and metabolomics profiling may provide insights into the consequences of abnormal buildup of glutamate in the cells.

Finally, how does the cyst(e)ine metabolic network crosstalk with other ferroptosis preventive pathways including the recently identified GCH1/BH4/DHFR axis^7,8^? How does this network shape the metabolic plasticity of cancer cells and contribute to the development of resistance to ferroptosis-inducing agents in vivo? Does the failure of any of these metabolic branches and nodes contribute to the induction of ferroptosis in organ damage and degenerative diseases beyond their roles in cancer? While these questions remain to be explored, insights from the present studies clearly imply new strategies to develop more effective ferroptosis-inducing therapies for the benefit of cancer patients.
