# Author Correction: Multifaceted mechanisms mediating cystine starvation-induced ferroptosis

**DOI:** 10.1038/s41467-023-36659-x

**Published:** 2023-02-22

**Authors:** Zhennan Shi, Nathchar Naowarojna, Zijian Pan, Yilong Zou

**Affiliations:** 1 Westlake Four-Dimensional Dynamic Metabolomics (Meta4D) Lab, Westlake Laboratory of Life Sciences and Biomedicine, Hangzhou, Zhejiang China; 2 School of Life Sciences, Westlake University, Hangzhou, Zhejiang China; 3 Institute of Biology, Westlake Institute of Advanced Studies, Hangzhou, Zhejiang China

**Keywords:** Lipid peroxides, Cancer metabolism, Necroptosis, Target identification

Correction to: *Nature Communications* 10.1038/s41467-021-25159-5, published online 09 August 2021

The original version of this Article contained an error in Ref. 11, which incorrectly gave the reference as: Zhang, Y. et al. mTORC1 couples cyst(e)ine availability with GPX4 protein synthesis and ferroptosis regulation. *Nat. Commun*. **12**, 1–14 (2021). The correct reference for Ref. 11 is: Zhang, Y. et al. mTORC1 couples cyst(e)ine availability with GPX4 protein synthesis and ferroptosis regulation. *Nat. Commun*. **12**, 1589 (2021). This has been corrected in the PDF and HTML versions of the Article.

